# Extraction of Ibuprofen from Natural Waters Using a Covalent Organic Framework

**DOI:** 10.3390/molecules25143132

**Published:** 2020-07-08

**Authors:** Soraia P. S. Fernandes, Abdelkarim Mellah, Petr Kovář, Marisa P. Sárria, Milan Pšenička, Harik Djamila, Laura M. Salonen, Begoña Espiña

**Affiliations:** 1 International Iberian Nanotechnology Laboratory (INL), Avenida Mestre José Veiga, 4715-330 Braga, Portugal; soraia.fernandes@inl.int (S.P.S.F.); mellah.abdelkarim@crstra.dz (A.M.); vmarisapassos@gmail.com (M.P.S.); 2 Organic Chemistry, Natural Products and Food Stuffs Research Unit (QOPNA), Department of Chemistry, University of Aveiro, Campus Universitário de Santiago, 3810-193 Aveiro, Portugal; 3 National Polytechnic School, Environmental Engineering Department, Laboratory of Sciences and Environmental Techniques, 10 Avenue Hacen Badi, BP182 El Harrach, Algiers 16200, Algeria; djamila.harik@g.enp.edu.dz; 4 Centre for Scientific and Technical Research on Arid Regions Omar El Bernaoui—CRSTRA, University Campus, Med Kheider BP 1682 R.P, Biskra 07000, Algeria; 5 Faculty of Mathematics and Physics, Charles University, Ke Karlovu 3, 121 16 Prague, Czech Republic; kovar@karlov.mff.cuni.cz (P.K.); milan.psenicka@centrum.cz (M.P.)

**Keywords:** covalent organic frameworks, pharmaceutical pollutants, adsorption, environmental water samples

## Abstract

Ibuprofen is one of the most widely used pharmaceuticals, and due to its inefficient removal by conventional wastewater treatment, it can be found in natural surface waters at high concentrations. Recently, we demonstrated that the TpBD-(CF_3_)_2_ covalent organic framework (COF) can adsorb ibuprofen from ultrapure water with high efficiency. Here, we investigate the performance of the COF for the extraction of ibuprofen from natural water samples from a lake, river, and estuary. In general, the complexity of the natural water matrix induced a reduction in the adsorption efficiency of ibuprofen as compared to ultrapure water. The best performance, with over 70% adsorption efficiency, was found in lake water, the sample which featured the lowest pH. According to the theoretical calculations, ibuprofen more favorably interacts with the COF pores in the protonated form, which could partially account for the enhanced adsorption efficiency found in lake water. In addition, we explored the effect of the presence of competing pharmaceuticals, namely, acetaminophen and phenobarbital, on the ibuprofen adsorption as binary mixtures. Acetaminophen and phenobarbital were adsorbed by TpBD-(CF_3_)_2_ with low efficiency and their presence led to an increase in ibuprofen adsorption in the binary mixtures. Overall, this study demonstrates that TpBD-(CF_3_)_2_ is an efficient adsorbent for the extraction of ibuprofen from natural waters as well.

## 1. Introduction

The increased release of pharmaceutical products into water, mainly through human and veterinary consumption, but also by the pharmaceutical industry, forced the World Health Organization to include these compounds in the list of emerging contaminants for regulation [1,2,3]. So far, a maximum level of 10 ng L^−1^ for the presence of pharmaceuticals in natural waters was established to avoid environmental and health risks [4]. Ibuprofen is not only the most widely used pharmaceutical compound, but also the most frequently found in natural and drinking water worldwide [2,3,5,6]. According to NORMAN and KNAPPE European projects, which compiled data from 117 studies on the presence of pharmaceuticals and personal care products (PPCPs) in wastewater from Europe, Brazil, and North America, ibuprofen is among the PPCPs with the highest concentrations in effluents [7]. This implies that not only is the use of ibuprofen extensive, but also conventional wastewater treatment is not efficient for its removal.

Among the wide variety of reported techniques for the extraction of contaminants from water and wastewater, adsorption is the most promising one, owing to its simplicity, low cost, and possibility of reusing the selected adsorbent [8]. The adsorption process can be affected by the nature of the adsorbent, but also by the physicochemical properties of the water matrix, such as temperature, pH, and salinity [8]. Ubiquitous in the water environment, natural organic matter (NOM), which is composed of a complex mixture of organic compounds, such as humic and fulvic acids, can also influence the adsorption process [9].

Covalent organic frameworks (COFs) [10,11] are fully organic, crystalline nanoporous materials, which feature large surface areas, tunable pore size and functionality, and high thermal and chemical stability. Recently, COFs have raised increasing interest for the extraction of different organic contaminants from water [12,13,14], such as biotoxins [15,16], perfluoroalkyl compounds [17], polycyclic aromatic hydrocarbons [18], and pharmaceutical pollutants [19,20,21]. In addition, COFs are being explored for membrane-based water treatment [22,23,24,25].

**Figure 1:**
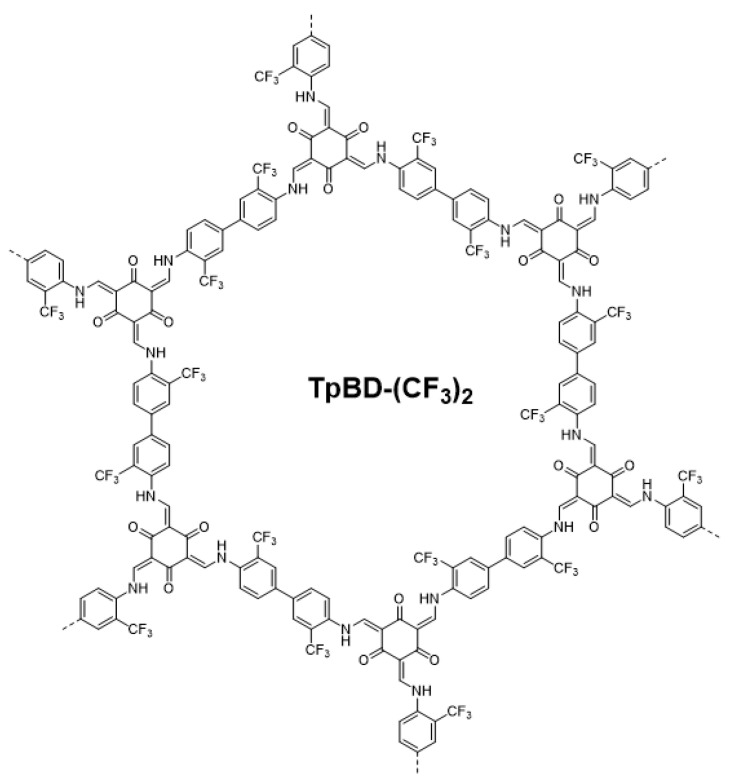
The pore structure of TpBD-(CF_3_)_2_.

Previously, we showed that TpBD-(CF_3_)_2_ COF (Figure 1) featured a marked preference for adsorbing lipophilic pharmaceuticals ibuprofen and diclofenac from ultrapure water, whereas hydrophilic acetaminophen and ampicillin were adsorbed far less efficiently [19]. In addition, an enhancement of ibuprofen adsorption efficiency was observed when moving to acidic pH, which was attributed mainly to increased lipophilicity upon protonation of the ibuprofen molecules [19].

Herein, we report the capacity of TpBD-(CF_3_)_2_ to extract pharmaceutical pollutant ibuprofen (Table 1) from natural water samples from the river, estuary, and lake collected in Northern Portugal. Theoretical calculations were conducted to gain insight into the differences in adsorption efficiencies found in the different water samples. Finally, the influence of coexisting molecules on ibuprofen adsorption was studied in binary mixtures of the compound with acetaminophen and phenobarbital.

## 2. Results and Discussion

### 2.1. Synthesis and Characterization of TpBD-(CF_3_)_2_

TpBD-(CF_3_)_2_ [19] was prepared by solvothermal synthesis from triformylphloroglucinol (Tp) and 3,3′-bis(trifluoromethyl)benzidine (BD-(CF_3_)_2_) in a mixture of mesitylene and 1,4-dioxane with aqueous 6 M acetic acid as catalyst at 120 °C for 3 days (for details, see the Supplementary Material, Section 2). An ordered porous material was obtained, as demonstrated by powder X-ray diffraction (Figure S1), with three main reflections at 2θ = 3.6°, 6.19°, and 25.3°. Nitrogen sorption measurements at 77 K gave a type I isotherm (Figure S2) with a Brunauer–Emmett–Teller surface area of 1090 m^2^ g^−1^ (Figure S3) and a pore volume of 0.50 cm^3^ g^−1^. Pore size distribution calculated using quenched-solid density functional theory (QSDFT) showed a maximum at 1.1 nm (Figure S4).

### 2.2. Extraction of Ibuprofen by TpBD-(CF_3_)_2_ in Natural Waters (Lake, River, and Estuary)

In order to study the capacity of TpBD-(CF_3_)_2_ to adsorb ibuprofen from natural water, we collected samples from lake, river, and estuary waters in the region of Viana do Castelo, Portugal. These samples feature different physical-chemical properties (Table S1). The pH of the water samples was found to vary from slightly acidic in lake water with pH 6.5 to slightly alkaline in the river and estuary waters with pH 7.7 and 7.8, respectively. High contents of ions such as calcium and magnesium were measured in lake water giving a total hardness GH of 8°, corresponding to a concentration of about 143 mg L^−1^, whereas in river water the value was below 1°. On the other hand, in river and estuary water, the content of carbonate ions, KH, detected was 7°, 125 mg L^−1^, which is 40% more than that registered in the lake water, giving an explanation to its lower pH.

To determine the adsorption efficiency of TpBD-(CF_3_)_2_ towards ibuprofen in lake, river, and estuary waters, adsorption experiments were performed over 2 h to ensure that equilibrium was reached, at 21 °C under constant shaking (1400 rpm), at a TpBD-(CF_3_)_2_ concentration of 330 mg L^−1^. The natural water samples were spiked with ibuprofen at concentrations of 50 µM and 100 µM, the latter corresponding to the solubility limit of the compound in water. These concentrations, despite being much higher than expected of a contamination with pharmaceuticals in nature, where values between few ng L^−1^ to µg L^−1^ in locations close to wastewater effluents can be observed [2,31], will allow us to compare the maximum capacity of the adsorbent previously observed in ultrapure water with the results obtained in this study.

At 100 µM, the adsorption capacities, *q_t_*, of TpBD-(CF_3_)_2_ for ibuprofen were found to be 42, 27, and 14 mg g^−1^ in lake, river, and estuary water, respectively (Table 2). By comparison, in our previous study in ultrapure water, adsorption capacity in equilibrium, *q_e_*, of 119 mg g^−1^ was found with a lower COF loading of 100 mg L^−1^ [19], corresponding to decreases of 65%, 77%, and 88%, for lake, river, and estuary waters, respectively. Such a reduction in adsorption capacity can be expected due to the complexity of natural water samples and could stem from the different physical-chemical parameters of the water samples and the presence of dissolved organic matter or competing molecules [9,32]. Previous studies with COFs have indicated that the presence of humic acid can severely affect the extraction efficiency of organic contaminants, with a reduction of adsorption efficiency up to 40% [18].

**Figure 2:**
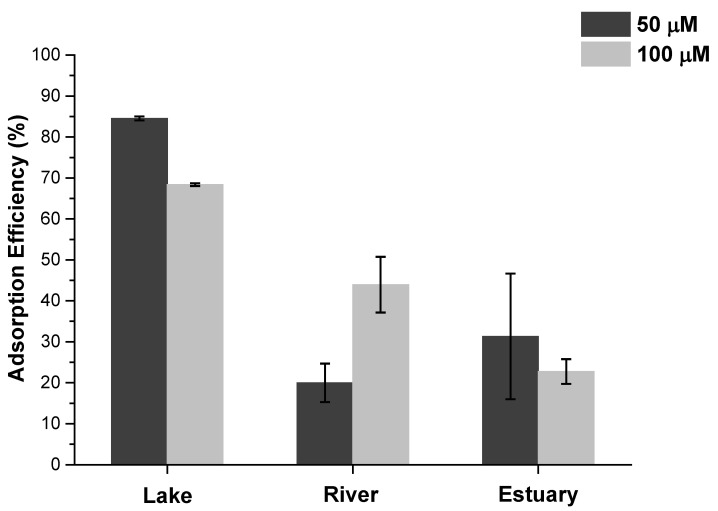
Adsorption efficiency (%) of ibuprofen at concentrations of 50 µM and 100 µM by TpBD-(CF_3_)_2_ (*C*_0_ = 330 mg L^−1^) in natural water samples collected from a lake, river, and estuary.

The highest adsorption efficiency, 85%, was found in lake water (Figure 2), whereas in river and estuary water the efficiency was below 50%. This could be due to the lower pH of lake water as compared to the other water samples. Previously, we found that adsorption of ibuprofen was enhanced by 25% when moving from neutral pH to pH 2 [19], which was attributed to the higher lipophilicity of the compound at lower pH upon protonation of the carboxylic acid moiety. With ibuprofen p*K*_a_ of 5.2, approximately 5% and 0.3% of the molecules are protonated at pH 6.5 and 7.7, respectively [27], which could partially explain the increased adsorption capacity in lake water. On the other hand, the amount of organic matter can be expected to be higher in lake and estuary waters as compared to river water, which could also affect the adsorption of ibuprofen. Additionally, the higher salinity of estuary water as compared to river or lake water can hinder the adsorption of ibuprofen, as demonstrated in a recent study [20], where the presence of CaCl_2_ and NaCl was found to significantly lower the adsorption capacity of a COF towards diclofenac, which was attributed to the cations competing for the adsorption sites with the pharmaceutical. However, a limited influence of this parameter was expected as the samples from estuary were collected from the first 1 m in the water column, which is mainly constituted by the river water. However, the day before the collection of samples a strong precipitation was registered, opening the possibility to the influence of organic matter and other ions from the outflow in the matrix effect [33].

### 2.3. Theoretical Calculations of the Interactions of Ibuprofen with TpBD-(CF_3_)_2_

#### 2.3.1. Models without Water

In order to gain insight into the differences of affinity of ibuprofen to TpBD-(CF_3_)_2_ in the natural water samples, we carried out theoretical calculations (for details, see Section 3.8.). Both protonated and deprotonated forms of ibuprofen were calculated to shed light on the differences in the adsorption efficiencies at different pH.

**Figure 3:**
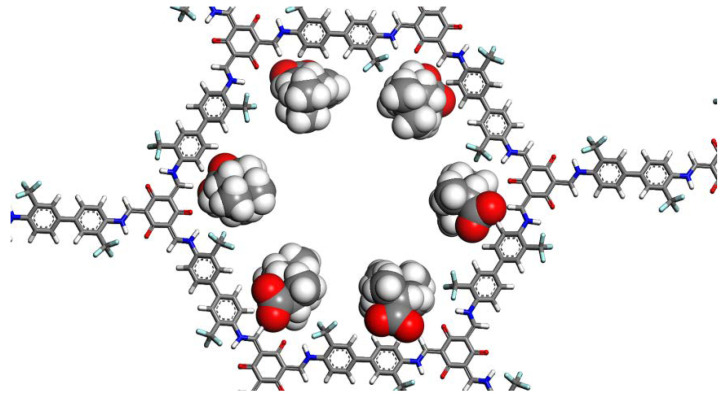
A view along the covalent organic framework (COF) pore axis showing the orientations of six deprotonated ibuprofen molecules.

In vacuo, both forms tend to adopt a very similar orientation in the COF pore, as seen in Figure 3 with six molecules with a supercell containing one entire pore. The longitudinal axes of the molecules are mostly oriented along the COF pore axis and can also adopt a tilted arrangement with respect to the COF pore axis during the simulation. The binding area extends over the oxygen atoms of Tp and neighboring CF_3_ and NH moieties of the COF, with the carboxyl group located between the COF layers (Figure S13).

#### 2.3.2. Models with Water

Next, we created models with water containing two ibuprofen molecules in protonated or deprotonated form and located them on the COF surface or in the COF pore. The results showed that in all cases the molecules had the tendency to move into the COF pore. Thereafter, models were created by locating two ibuprofen molecules in protonated and deprotonated form each in the COF pore. This corresponds to a situation found at pH 5.2, wherein half of the molecules are protonated and the other half deprotonated (Figure S14).

**Figure 4:**
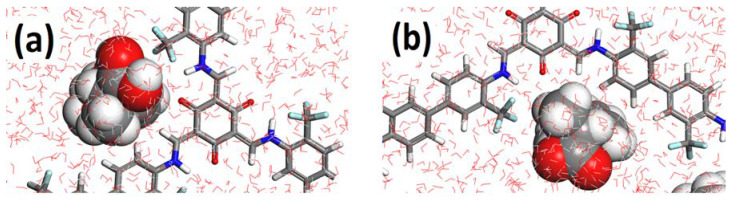
A view along the COF pore axis showing the orientations of protonated (**a**) and deprotonated (**b**) ibuprofen in water environment.

The representative models containing two ibuprofen molecules in the pore are shown in Figure 4. Both protonated (Figure 4a) and deprotonated (Figure 4b) forms exhibit a very similar trend: they are located in the same binding area, in the corner of the COF pore formed by Tp, as in the case of models without water, and their longitudinal axes are nearly parallel to the COF pore axis. However, the carboxyl group of deprotonated ibuprofen faces the water environment, whereas protonation causes the molecule to flip and interact via hydrogen bonds with O atoms of Tp and NH moieties or form short contacts with the fluorine atoms of the CF_3_ moiety. The average distance between the hydrogen atoms of the carboxyl group and a fluorine or O atom of the COF is 2.7 Å.

In the models with two ibuprofen molecules in the COF pore the average interaction per one ibuprofen molecule between the deprotonated form and the COF structure was −19 kcal mol^−1^, whereas for the protonated form a slightly enhanced value of −21 kcal mol^−1^ was found. The main driving force was found to be van der Waals interactions with ca. 90%. A large difference in the interactions between water and the two ibuprofen forms was observed. In the protonated form, the interaction energy with water was comparable to that with the COF; i.e., −20 kcal mol^−1^. However, a dramatic enhancement of interaction energy with water was found for the unprotonated form, giving an average value of −160 kcal mol^−1^ per ibuprofen molecule. In the model at pH 5.2, this interaction was 7–8 times stronger compared to that of the protonated form. Therefore, ibuprofen in the deprotonated form has a much higher tendency to be solvated by water than to be adsorbed on the COF, which could account for the experimental results obtained with the natural water samples. With decreasing pH, the quantity of protonated ibuprofen increases, leading to enhanced tendency of the pharmaceutical to be adsorbed within the COF pores.

### 2.4. Comparative Extractions of Ibuprofen, Phenobarbital, and Acetaminophen from Lake Water by TpBD-(CF_3_)_2_

We next tested the adsorption efficiency of TpBD-(CF_3_)_2_ for other pharmaceuticals for comparison, and selected phenobarbital and acetaminophen due to their broad use and different physico-chemical properties (Figure 1). Phenobarbital is still one of the most widely used antiepileptic drugs worldwide [34] and one of the active ingredients of the anticonvulsant primidone. Phenobarbital has been found in urban wastewater effluents at 0.09–0.21 μg L^−1^, and at concentrations of ≤0.05 μg L^−1^ in downstream surface waters [35]. On the other hand, acetaminophen is also frequently found in natural and drinking water worldwide, reaching concentrations of over 500 ng L^−1^ in river waters [2,3,5,6]. In our previous study, acetaminophen was not efficiently adsorbed by TpBD-(CF_3_)_2_, which we attributed to its higher hydrophilicity (log*D*_6.0_ = 0.34) as compared to ibuprofen (log*D*_6.0_ = 2.12) [26]. Phenobarbital, on the other hand, is more lipophilic than acetaminophen, but less than ibuprofen (log*D*_6.0_ = 1.66, Figure 1) [26].

**Figure 5:**
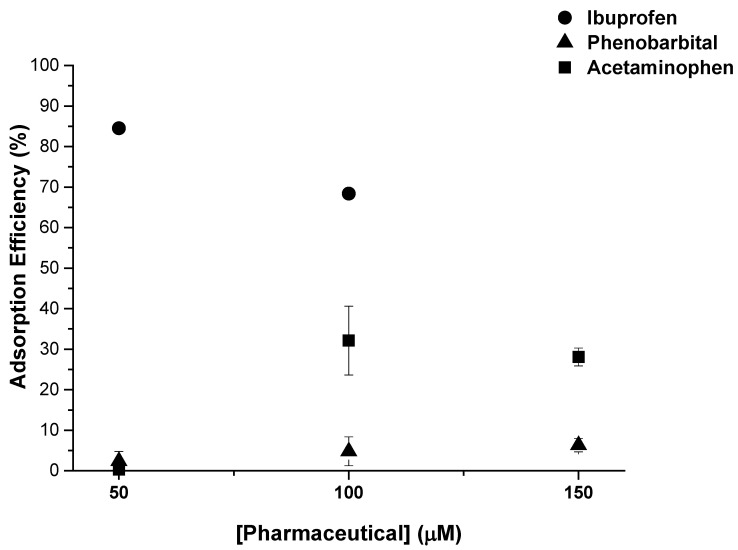
Adsorption efficiency (%) of ibuprofen at concentrations of 50 and 100 µM, and acetaminophen and phenobarbital at concentrations of 50, 100, and 150 µM by TpBD-(CF_3_)_2_ (*C*_0_ = 330 mg L^−1^) in lake water. The experiment was performed in duplicate.

Lake water was spiked with 50, 100, or 150 µM of acetaminophen or phenobarbital and exposed to TpBD-(CF_3_)_2_ at a concentration of 330 mg L^−1^ at 21 °C for 2 h. As expected, very low adsorption efficiency was found for acetaminophen, with values of 0.3%, 32%, and 28% recorded for 50, 100, and 150 µM, respectively (Figure 5, squares). The adsorption capacity of 19 mg g^−1^, obtained at the highest concentration (Table 3), was similar to that found in our previous study, albeit with a lower COF loading [19]. Phenobarbital showed low adsorption efficiencies, around 5%, at all concentrations tested (Figure 5, triangles).

### 2.5. Extraction of Pharmaceuticals in Binary Mixtures from Lake Water by TpBD-(CF_3_)_2_

In order to study the influence of competing compounds on the adsorption capacity of TpBD-(CF_3_)_2_ towards ibuprofen, we carried out adsorption experiments on binary mixtures. The total concentration of spiked pharmaceuticals was kept at 200 µM, using ratios of 50/150 and 100/100 µM for each binary mixture under the same incubation conditions as described above (for further details, see Section 3.7).

When ibuprofen was combined with either acetaminophen or phenobarbital at 50/150 µM ratio, the quantity of ibuprofen adsorbed by TpBD-(CF_3_)_2_ remained the same. The values of adsorption of acetaminophen and phenobarbital remained the same as in the individual adsorption tests (Table 3).

**Figure 6:**
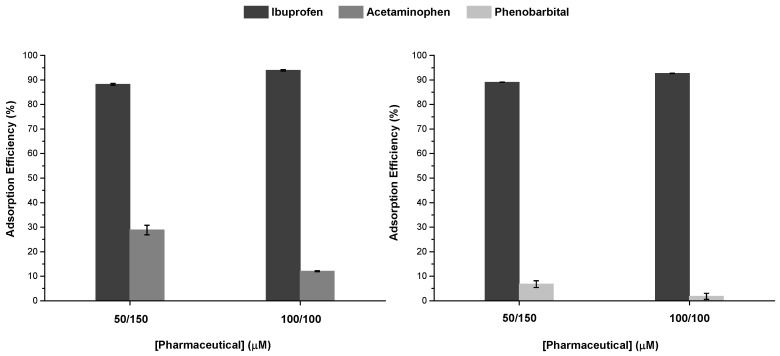
Adsorption efficiency (%) of binary mixtures of ibuprofen and acetaminophen or phenobarbital, at concentration ratios of 50/150 and 100/100 µM using TpBD-(CF_3_)_2_ (*C*_0_ = 330 mg L^−1^) as adsorbent in lake water samples.

At a 100/100 µM ratio, an increase of more than 10 mg g^−1^ was found for ibuprofen, whereas decreases were observed for both acetaminophen and phenobarbital of about 38% and 40%, respectively (Figure 6). The reduction can be explained by the competitive adsorption of ibuprofen, a compound with high affinity to the COF, which hinders the adsorption of the other two pharmaceuticals. Many previous studies have reported how the presence of competing molecules can enhance the uptake of an adsorbate with higher affinity for the adsorbent [36]. However, the differential interference exerted by the NOM and different ions present in the water sample on each pharmaceutical cannot be ruled out.

## 3. Materials and Methods

### 3.1. Chemicals and Instrumentation

Ultrapure water was produced by Milli-Q Advantage A10 system (Millipore, Molsheim, France, resistivity: 18.2 MΩ cm^−1^). Acetonitrile and methanol of high-performance liquid chromatography (HPLC) grade, and trifluoroacetic acid (TFA) 99% were purchased from Fisher Scientific, Porto Salvo, Portugal. Ibuprofen, acetaminophen, and phenobarbital were purchased from Sigma-Aldrich (Algés, Portugal) with a purity of 99.9%.

### 3.2. Water Samples’ Locations and Physical-Chemical Parameters

All water samples were collected in Northern Portugal on the 6th of May, 2017, between 15 and 16 h. High tide was at 13.13 h and low tide at 19.16 h; however, a limited influence of seawater was expected as the samples were collected in the first 1 m of the water column. River water sample was collected from Lima river in Viana do Castelo (41°41′17.7′’ N 8°47′23.9′’ W), lake water sample from lake of São Pedro de Arcos in Ponte de Lima (41°45′52.5′’ N 8°38′13.60′’ W), and estuary water from Lima river near the mouth of the river (41°40′58.14′’ N 8°49′35.67′’ W). pH was measured using a benchtop pHmeter (SevenCompact, Mettler Toledo) with an accuracy of ± 0.002. All other physical-chemical parameters of water were measured using the Sera Aqua-Test (Table S1).

### 3.3. Quantification of Pharmaceutical Products by HPLC

Agilent Technologies (Waldbronn, Germany) 1200 series HPLC system was used for the identification and quantification of the pharmaceuticals in natural water samples of the lake, river, and estuary. HPLC was equipped with a pump module, a vacuum degasser, an autosampler, a thermostatted column compartment, and a diode-array detector (DAD). Data acquisition was performed by Agilent’s ChemStation software, version 1.9.0. Reverse phase analysis was carried out with a Kinetex EVO C-18 column purchased from Phenomenex (Madrid, Spain) (reversed phase, particle size of 2.6 µm, pore size of 100 Å, length 100 mm, internal diameter 4.6 mm) at 20 °C. Mobile phase consisted of 0.1% *v*/*v* TFA in ultrapure water (phase A) and 0.1% *v*/*v* TFA in acetonitrile (phase B). A gradient ratio of 98% of phase A and 2% of phase B was employed for 6 min. Then, during 5 min a gradient ratio of 70% phase A and 30% phase B was applied. The injection volume was set to 20 µL and a flow rate of 1.25 mL min^−1^ was maintained during the whole analysis. Detection of ibuprofen was performed at λ = 220 nm and a retention time of around 9.5 min; acetaminophen at λ = 243 nm with a retention time of around 2.8 min; and phenobarbital at λ = 210 nm with a retention time of around 6.1 min.

### 3.4. Stock Solutions

Stock solutions of the pharmaceuticals were prepared in methanol, to ensure the solubility of pharmaceuticals, with final concentrations of 1.03 and 2.06 g L^−1^ for ibuprofen; 0.76, 1.51, and 2.27 g L^−1^ for acetaminophen; and, 1.16, 2.32, and 3.48 g L^−1^ for phenobarbital. COF stock solutions were prepared in lake, river, and estuary waters with a final concentration of 336 mg L^−1^.

### 3.5. Calibration Curves of Ibuprofen, Acetaminophen, and Phenobarbital in Ultrapure Water

Calibration curves of ibuprofen, acetaminophen, and phenobarbital were prepared with concentrations between 0 and 30 mg L^−1^ in ultrapure water (pH 6–7). The samples were analyzed by HPLC-DAD as described in Section 3.3. The area of the chromatographic peak typical of each pharmaceutical was determined. The peak area (mAu s^−1^) versus pharmaceutical concentration was plotted and a linear regression was applied (Figures S10–S12).

### 3.6. Extraction of Ibuprofen from Lake, River, and Estuary Waters, and Acetaminophen and Phenobarbital from Lake Water by TpBD-(CF_3_)_2_

Adsorption experiments were performed using lake, river, and estuary natural water samples, which were spiked individually with ibuprofen, acetaminophen, and phenobarbital stock solutions. Concentration of methanol in water mixtures was always below 1% of total volume. For adsorption of ibuprofen, TpBD-(CF_3_)_2_ COF dispersions of 330 mg L^−1^, prepared in each natural water sample, were spiked with ibuprofen concentrations of 0.05 and 0.10 mmol L^−1^, resulting in a final volume of 600 µL. For acetaminophen and phenobarbital adsorption, TpBD-(CF_3_)_2_ COF dispersions of 330 mg L^−1^, prepared in lake water samples, were spiked with acetaminophen or phenobarbital concentrations of 0.05, 0.10, and 0.15 mmol L^−1^, resulting in a final volume of 600 µL. The mixtures were prepared as duplicates and incubated under constant shaking at 1400 rpm and 21 ± 2 °C for 2 h. After this time, the supernatant was isolated by centrifugation (15000 rpm, 21 °C, 15 min) and filtered through a 0.22 µm polysulfone syringe filter. Supernatant was analyzed by HPLC-DAD to quantify the amount of pharmaceutical remaining in the solution after adsorption. The characteristic retention time of each pharmaceutical allowed their identification. The surface area of the chromatographic peak was determined using the software OpenLab. The amount of the pharmaceutical adsorbed onto the COF, *q_t_* (mg g^−1^), was calculated using the following equation:

$$q_{t}=\left(C_{0}-C_{t}\right)\frac{V}{m}$$

where *C*_0_ and *C_t_* (mg L^−1^) are the concentrations of the pharmaceutical in the initial solution and after 2 h of adsorption, respectively, *V* (L) is the volume of the samples, and *m* (mg) is the mass of the adsorbent.

### 3.7. Adsorption Experiments of Binary Mixtures of Ibuprofen and Acetaminophen or Phenobarbital in Lake Water by TpBD-(CF_3_)_2_

Adsorption experiments were performed using lake water samples, which were spiked with mixtures of ibuprofen with acetaminophen or phenobarbital, in the ratios of 0.05 mmol L^−1^ of ibuprofen and 0.15 mmol L^−1^ of acetaminophen or phenobarbital (50/150 µM) and 0.10 mmol L^−1^ of ibuprofen and 0.10 mmol L^−1^ of acetaminophen or phenobarbital (100/100 µM). For the adsorption, TpBD-(CF_3_)_2_ dispersions of 330 mg L^−1^, prepared in lake water samples, were spiked with known concentrations in 50/150 µM ratio or 100/100 µM ratio of ibuprofen and acetaminophen or phenobarbital, respectively, resulting in a final volume mixture of 600 µL. The adsorption experiment and analysis were carried out as described above.

### 3.8. Theoretical Calculations

The structure of TpBD-CF_3_ was previously described [19]. The cell parameters were *a* = *b* = 28.67 Å, *c* = 4.25 Å, α = β = 90°, γ = 120 °; the space group was P6. A 3D periodic box with space group P1 and the dimensions 1a × 1b × 1C was created from the original cell; C ≈ 80 Å. The box contained six COF layers of a total thickness of ca. 28 Å, simulating the COF surface and a part of its pore. The ibuprofen molecule was built in a Forcite module of Materials Studio modelling environment, version 8 [37] and its geometry was optimized. Based on the experimental data, both deprotonated and protonated forms of ibuprofen were used. To better understand and describe the behavior of ibuprofen, a series of different calculations was carried out: (i) COF models with the ibuprofen molecules on the COF surface, and (ii) COF models with the ibuprofen molecules in the pore. In both cases, protonated or deprotonated form of the pharmaceutical, and the mixture thereof were used. At first, 1 ns dynamics simulations at 298 K in an NVT statistical ensemble (N—constant number of atoms, V—constant volume, T—constant temperature) were carried out without water to find the binding sites of ibuprofen on the COF structure. The obtained models were used for subsequent calculations. A corresponding amount of water to keep the density of 1000 kg m^−3^ was added into the box; the geometry of these models was optimized and pre-equilibrated in Materials Studio software. After this, dynamics simulations were carried out in an NVT statistical ensemble in Lammps simulation package [38]. At first, the systems were heated up to 500 K for 1 ns and after cooling down 2 ns dynamics simulations at 298 K were carried out. The snapshots were collected every 2000 steps and those from the last 1 ns of the simulation were used for the analysis. In all cases one dynamic step was 1 fs, the atomic positions of COF were kept fixed, and all other atomic positions were variable. The calculations were done in pcff force field [39]; the atomic charges were assigned by the Compass force field [40]. The electrostatic interactions were calculated by PPPM method and van der Waals interactions were calculated by Lennard–Jones potential with a cut-off of 12 Å.

## 4. Conclusions

In this study, TpBD-(CF_3_)_2_ COF, previously demonstrated as an excellent adsorbent for ibuprofen in ultrapure water, was shown to be an efficient adsorbent of ibuprofen in natural waters as well. The physical-chemical properties of the water determined the adsorption efficiency, and pH was identified as the key parameter. In agreement with the theoretical calculations, which showed the protonated form of ibuprofen to preferentially interact with the COF pores, the highest affinity of the COF adsorbent was found in lake water that featured slightly acidic pH. Finally, the results of competitive adsorption with other commonly found pharmaceuticals in waters, acetaminophen and phenobarbital, tested as binary mixtures, showed that the adsorption efficiency of ibuprofen is not hindered, but enhanced by the presence of other lower affinity pharmaceuticals.

## Figures and Tables

**Table 1:** Properties of the studied pharmaceuticals. Dimensions of pharmaceuticals were obtained by measuring the furthest distances of C, N, or O atoms from the X-ray crystal structures obtained from the Cambridge Structural Database (CSD) with the following codes: ^a^ CCDC 1041382, ^b^ CCDC-1149948, ^c^ CCDC-150969.

	Ibuprofen	Phenobarbital	Acetaminophen
	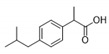	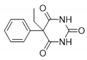	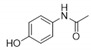
Dimensions [Å]	^a^ 8.8 × 4.5 × 2.4	^b^ 6.5 × 4.5 × 4.3	^c^ 7.9 × 2.4 × 0.8
log*D*_6.0_ [26]	2.12	1.66	0.34
p*K*_a_	5.2 [27]	7.3 [28]	9.5 [29]
Water solubility [g L^−1^]	0.021 [30]	1 [28]	14 [30]

**Table 2:** Adsorption capacity, *q_t_* (mg g^−1^), of ibuprofen by TpBD-(CF_3_)_2_ at 21 °C; *t* = 2 h; covalent organic framework (COF) concentration of 330 mg L^−1^.

		Lake	River	Estuary
	[µM]	*q_t_* (mg g^−1^)	*q_t_* (mg g^−1^)	*q_t_* (mg g^−1^)
**Ibuprofen**	50	26.2 ± 0.2	6.2 ± 2.1	9.7 ± 9.3
	100	42.3 ± 0.3	27.2 ± 6.0	14.1 ± 2.6

**Table 3:** Adsorption capacity *q_t_* (mg g^−1^), of TpBD-(CF_3_)_2_ (*C*_0_ = 330 mg L^−1^) in binary mixtures of ibuprofen and acetaminophen or phenobarbital (concentration ratios of 50/150 and 100/100 µM).

	Individual Pharmaceutical		Binary Mixture
**[µM]**	**Ibuprofen**	**Acetaminophen**	**Ibuprofen/Acetaminophen**
**50/150**	26.2 ± 0.2	19.1 ± 2.1	27.3 ± 0.2 / 19.6 ± 1.9
**100/100**	42.3 ± 0.3	14.6 ± 5.5	58.1 ± 0.3 / 5.5 ± 0.1
	**Ibuprofen**	**Phenobarbital**	**Ibuprofen/Phenobarbital**
**50/150**	26.2 ± 0.2	6.6 ± 2.4	27.6 ± 0.01 / 7.1 ± 2.0
**100/100**	42.3 ± 0.3	3.3 ± 3.5	57.3 ± 0.1 / 1.3 ± 1.2

## Supplementary Materials

The following are available online at https://www.mdpi.com/1420-3049/25/14/3132/s1: Section 2: COF synthesis, Figure S1: Powder X-ray diffraction pattern of TpBD-(CF_3_)_2_, Figure S2: Nitrogen adsorption (filled spheres) and desorption (hollow spheres) isotherm profile measured at 77 K of TpBD-(CF_3_)_2_, Figure S3: Multi-point BET plot and linear fit of TpBD-(CF_3_)_2_, Figure S4: Pore size distribution and cumulative pore volume profiles of TpBD-(CF_3_)_2_, Figures S5–S19: Chromatograms of the water samples; Figures S20–S22: Calibration curves, Figure S23: A view perpendicular to the COF pore axis showing the protonated ibuprofen in tilted and parallel orientation with respect to the COF pore axis, Figure S24: A view along the COF pore axis showing the orientations of deprotonated and protonated ibuprofen molecules in the model in a water environment.. In addition, a supplementary 3D file is provided to accompany Figure S23; Table S1: Physical-chemical parameters of river, lake and estuary water collected.
