# Supplementary material for: Extraction of Ibuprofen from Natural Waters Using a Covalent Organic Framework
Source: Molecules. 2020 Jul 8;25(14):3132. doi: 10.3390/molecules25143132 (PMC7397005; doi:10.3390/molecules25143132)
Supplement: Supplementary file 1 [file molecules-25-03132-s001.zip › molecules-851982 supplementary xml.docx]

# Supplementary Materials

Extraction of Ibuprofen from Natural Waters Using a Covalent Organic Framework

**Soraia P. S. Fernandes ^1,2,†^, Abdelkarim Mellah ^1,3,4,†^, Petr Kovář ^5^, Marisa P. Sárria ^1^,
Milan Pšenička ^5^, Harik Djamila ^3^, Laura M. Salonen ^1,^* and Begoña Espiña ^1,^***

^1^ International Iberian Nanotechnology Laboratory (INL), Avenida Mestre José Veiga, 4715-330 Braga, Portugal; soraia.fernandes@inl.int (S.P.S.F.); mellah.abdelkarim@crstra.dz (A.M.); vmarisapassos@gmail.com (M.P.S.)

^2^ Unidade de Investigação de Química Orgânica, Produtos Naturais e Agrolimentares (QOPNA), Department of Chemistry, University of Aveiro, Campus Universitário de Santiago, 3810-193 Aveiro, Portugal

^3^ National Polytechnic School, Environmental Engineering Department, Laboratory of Sciences and Environmental Techniques, 10 Avenue Hacen Badi, BP182 El Harrach 16200 Algiers, Algeria; djamila.harik@g.enp.edu.dz

^4^ Centre for Scientific and Technical Research on Arid Regions Omar El Bernaoui—CRSTRA,University campus, Med Kheider BP 1682 R.P Biskra 07000, Algeria

^5^ Charles University, Faculty of Mathematics and Physics, Ke Karlovu 3, Prague, 121 16, Czech Republic; milan.psenicka@centrum.cz

^†^ These authors contributed equally.

***** Correspondence: [laura.salonen@inl.int](mailto:laura.salonen@inl.int) (L.M.S.); [begona.espina@inl.int](mailto:begona.espina@inl.int) (B.E.); Tel.: + 351 253 140 112

1. Materials and Methods

Tp was synthesized following a literature-known procedure [1]. 3,3’‑Bis(trifluoromethyl)benzidine was purchased from Apollo Scientific, anhydrous 1,4‑dioxane from Acros Organics, and mesitylene 99% extra pure from Fisher Chemical. Aqueous acetic acid was prepared by dilution of commercial acetic acid, ACS reagent, ≥ 99.8% purchased from Sigma-Aldrich. *N*,*N*-Dimethylformamide HPLC grade and acetone 99.5% were purchased from Fisher Chemical and Honeywell, respectively, and used as received.

Powder X-ray diffraction (PXRD) measurements were carried out in reflection mode on a Bruker D8 Discover diffractometer with Ni-filtered Kα radiation (*λ* = 1.54060 Å) and a position-sensitive detector (LynxEye).

Nitrogen sorption measurements were performed using a Quantachrome Autosorb IQ2 automated analyzer. Prior to the measurements, samples were outgassed by heating to 120 °C (heating rate: 5 °C min^−^1, dwelling time: 720 min). Multipoint Brunauer–Emmett–Teller (BET) method using ASIQwin™ software was used to estimate the surface areas of the obtained powders. Pore size distributions were assessed using quenched-solid density functional theory (QSDFT) equilibrium model for slit pores (N_2_ at 77 K on carbon).

HPLC-DAD analysis was performed in an Agilent Technologies 1200 series high-performance liquid chromatograph coupled with a diode-array detector.

2. Synthesis of TpBD-(CF_3_)_2_

TpBD-(CF_3_)_2_ was prepared as described in our previous report.[2] Briefly, Tp (229 mg, 1.09 mmol, 1.0 equiv.) and 3,3’-bis(trifluoromethyl)benzidine (523 mg, 1.64 mmol, 1.5 equiv.) were placed in a 100 mL pressure tube (ACE glass, bushing type back seal, 17.8 cm x 38.1 mm), and 1,4-dioxane (4.5 mL) and mesitylene (4.5 mL) were added. The resulting suspension was sonicated at room temperature (r.t.) for 2 min to obtain a homogeneous mixture, and aqueous 6 M acetic acid (0.91 mL, 5.45 mmol) was added. The mixture was stirred at 120 °C for 3 d, cooled to RT, and filtered. The solid was washed with DMF (3 x 10 mL) until a colorless filtrate was observed, and then H_2_O (2 x 10 mL), acetone (3 x 10 mL), and CH_2_Cl_2_ (3 x 10 mL). The resulting solid was dried under high vacuum at 120 °C to give 696 mg of TpBD-(CF_3_)_2_ as yellow solid.

3. Adsorption Methodology

Adsorption experiments were performed as duplicates and standard deviation of the amount of pharmaceuticals adsorbed by COF, *q*t, was determined.

3.1. Extraction of Ibuprofen From Lake, River, and Estuary Water, and Acetaminophen and Phenobarbital from Lake Water by TpBD-(CF_3_)_2_

From ibuprofen stock solutions, 6 µL was withdrawn and added to a mixture of 594 µL of COF stock solution prepared in lake, river, and estuary water, respectively, to obtain a final pharmaceutical concentration of 0.05 or 0.10 mmol L^−^1.

Similarly, from acetaminophen and phenobarbital stock solutions, 6 µL were withdrawn and added to a mixture of 594 µL of COF stock solution prepared in lake water, to obtain final pharmaceutical concentrations of 0.05, 0.10, and 0.15 mmol L^−^1.

3.2. Adsorption Experiments of Binary Mixtures of Ibuprofen and Acetaminophen or Phenobarbital in Lake Water by TpBD-(CF_3_)_2_

For mixtures of ibuprofen with acetaminophen or phenobarbital in the ratios of 50/150 µM or 100/100 µM, 6 µL of ibuprofen stock solutions and 6 µL of acetaminophen or phenobarbital stock solutions were withdrawn and added to a mixture of 588 µL of COF stock solution prepared in lake water.


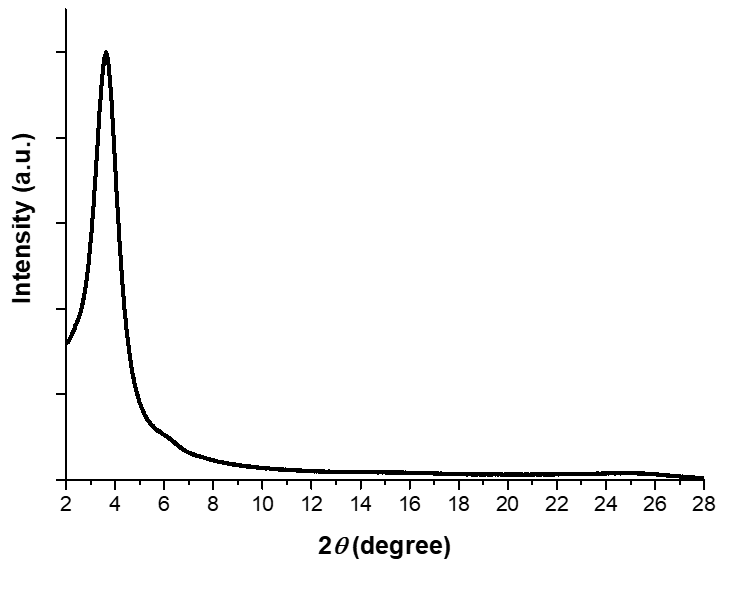
4. Powder X-Ray Diffraction

**Figure S1.** Powder X-ray diffraction pattern of TpBD-(CF_3_)_2_.

5. N_2_ Physisorption

**
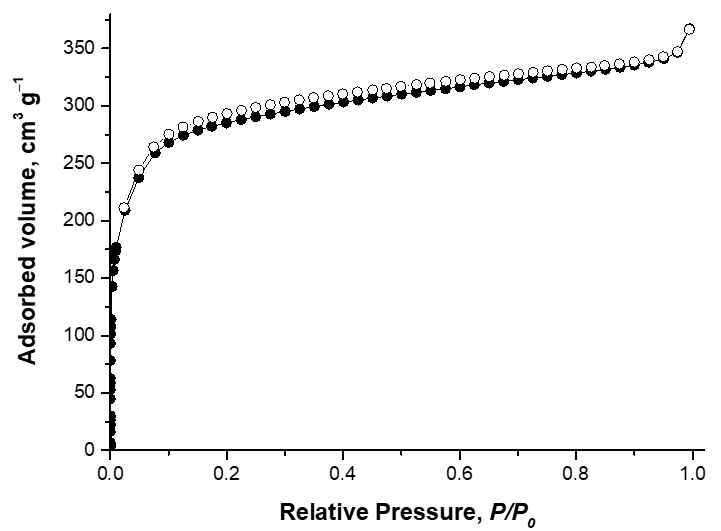
**

**Figure S2.** Nitrogen adsorption (filled spheres) and desorption (hollow spheres) isotherm profile measured at 77 K of TpBD-(CF_3_)_2_.

**Figure S3.** Multi-point BET plot and linear fit of TpBD-(CF_3_)_2_.


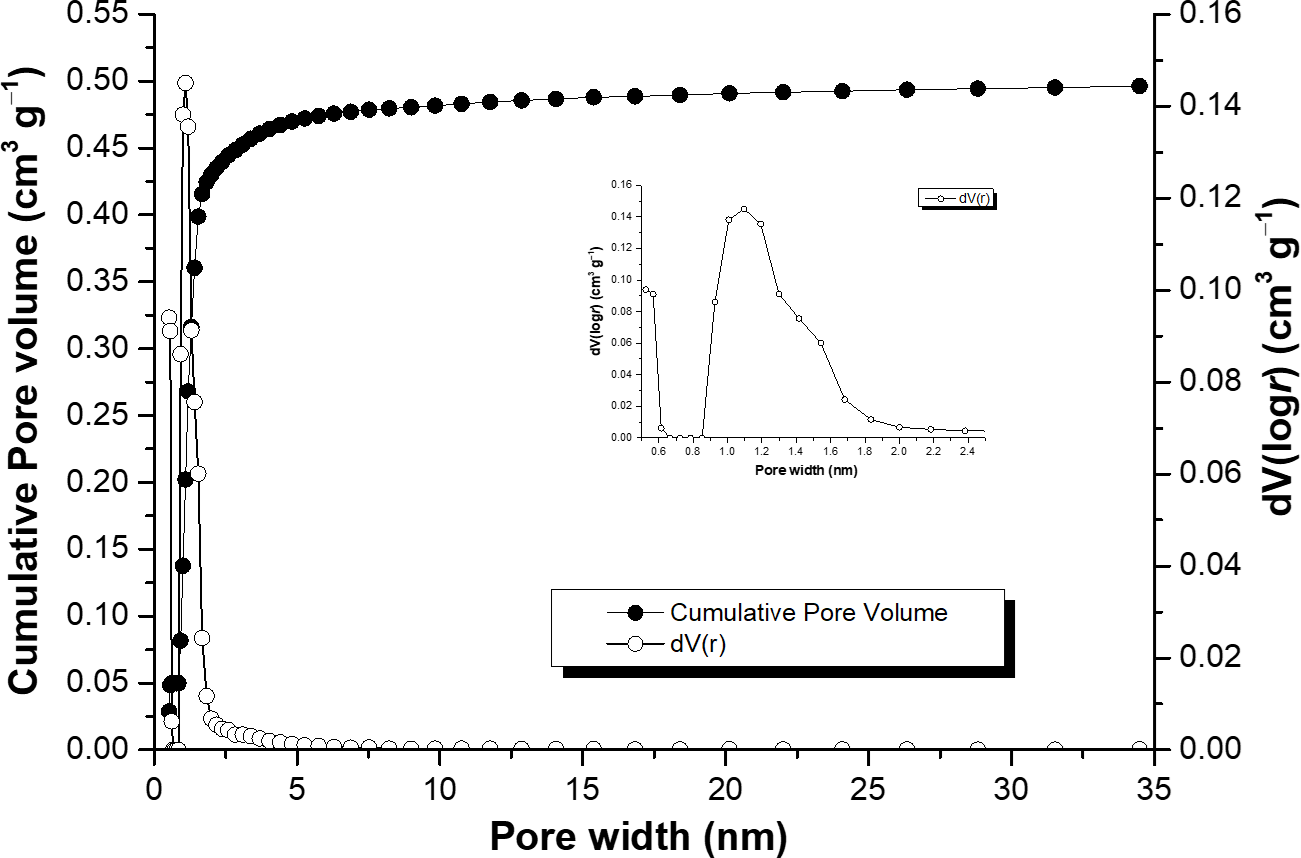


**Figure S4.** Pore size distribution (hollow spheres) and cumulative pore volume (filled spheres) profiles of TpBD-(CF_3_)_2_.

6. Physical-Chemical Parameters of Natural Water Samples

**Table S1.** Physical-chemical parameters of river, lake and estuary water collected. GH = total hardness; KH = carbonate hardness; LOD = limit of detection; n/a = not applicable.

| **Water sample** | **Lake** | **River** | **Estuary** |
| --- | --- | --- | --- |
| **GH (dGH)** | 8 | < LOD | n/a |
| **KH (dKH)** | 4 | 7 | 7 |
| **NH_4_ (mg L^−^1)** | 0.50 | < 0.50 | < 0.50 |
| **NH_3_ (mg L^−^1)** | < 0.003 | 0.03 | 0.03 |
| **NO_2_ (mg L^−^1)** | 0.00 | 0.00 | 1.00 |
| **NO_3_ (mg L^−^1)** | 0.00 | 0.00 | 10.00 |
| **PO_4_ (mg L^−^1)** | 1.00 | 2.00 | 1.00 |
| **Fe (mg L^−^1)** | 0.50 | 0.00 | 0.00 |

7. HPLC-DAD Chromatograms of Lake, River, and Estuary Natural Water Samples

The chromatograms of the natural water samples of lake, river, and estuary were obtained without spiking of pharmaceuticals, running at the wavelengths of analysis for ibuprofen (*λ* = 220 nm, retention time *t*r = 9.5 min), acetaminophen (*λ* = 243 nm, *t*r = 2.8 min), and phenobarbital (*λ* = 210 nm, *t*r = 6.1 min).


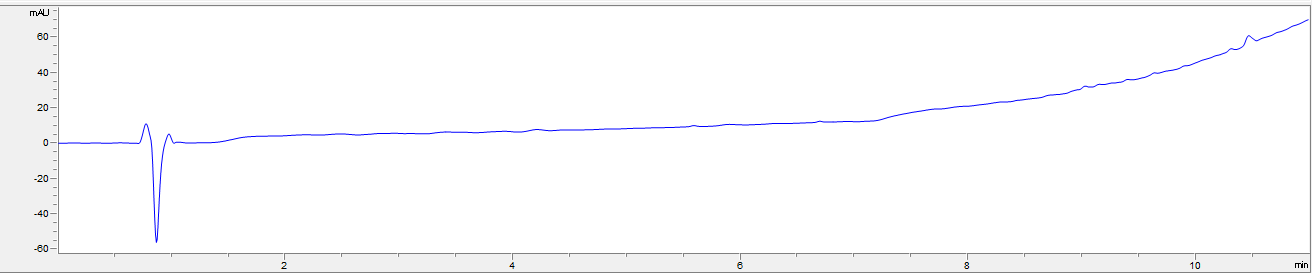


**Figure S5.** HPLC-DAD chromatogram for lake natural water sample (*λ* = 220 nm).


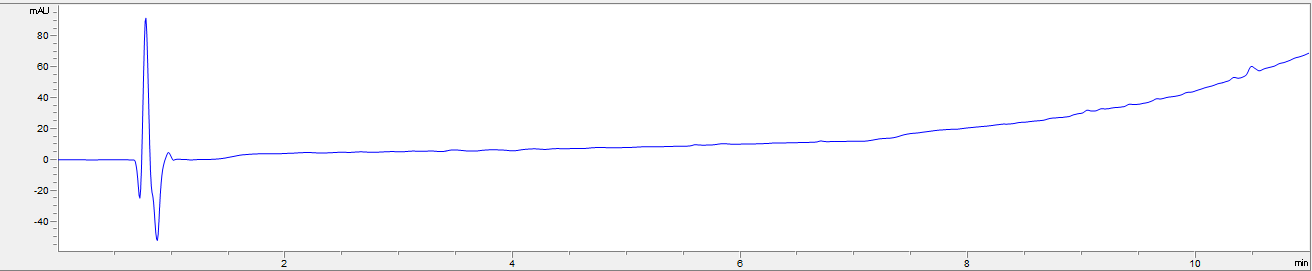


**Figure S6.** HPLC*-*DAD chromatogram obtained for river natural water sample (*λ* = 220 nm).


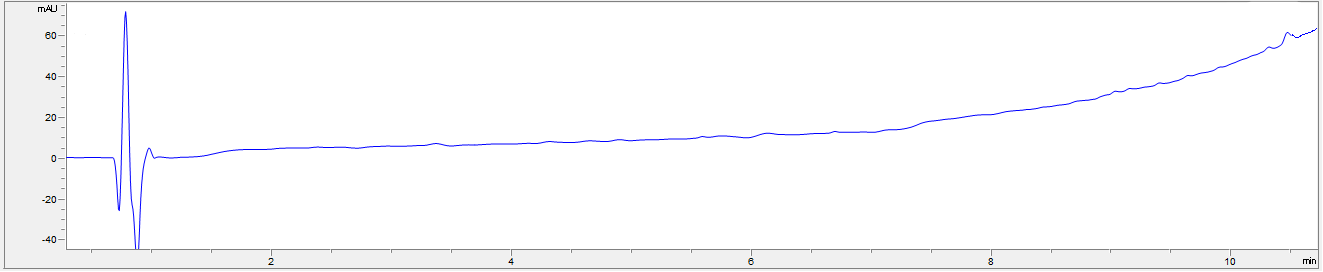


**Figure S7.** HPLC*-*DAD chromatogram obtained for estuary natural water sample (*λ* = 220 nm).


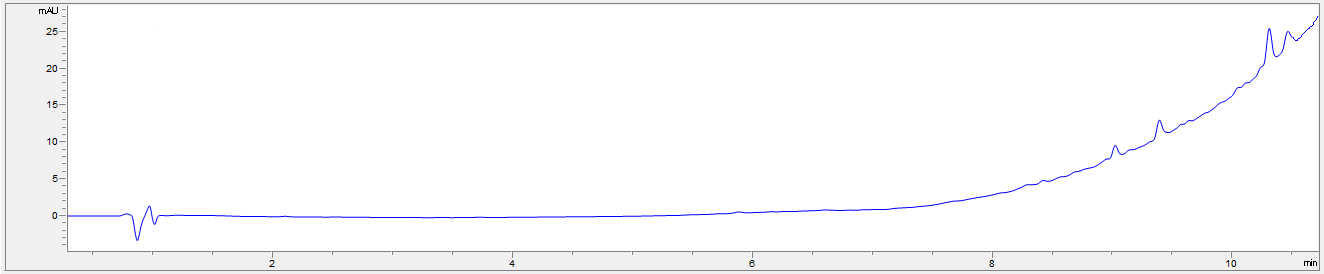


**Figure S8.** HPLC*-*DAD chromatogram obtained for lake natural water sample (λ = 243 nm).


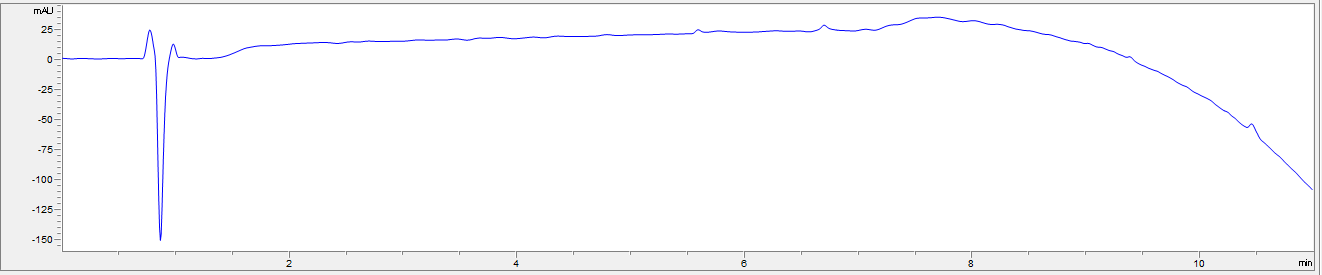


**Figure S9.** HPLC*-*DAD chromatogram obtained for lake natural water sample (*λ* = 210 nm).

The chromatograms of the natural water samples of lake, river, and estuary were obtained after spiking of pharmaceuticals running at the wavelengths of analysis for ibuprofen (*λ* = 220 nm, *t*r = 9.5 min), acetaminophen (*λ* = 243 nm, *t*r = 2.8 min), and phenobarbital (*λ* = 210 nm, *t*r = 6.1 min). *C*0 corresponds to the concentration of pharmaceutical spiked in natural water samples; *C*f corresponds to the concentration of pharmaceutical observed in natural water samples after spiking, calculated using the calibration curve of each pharmaceutical.


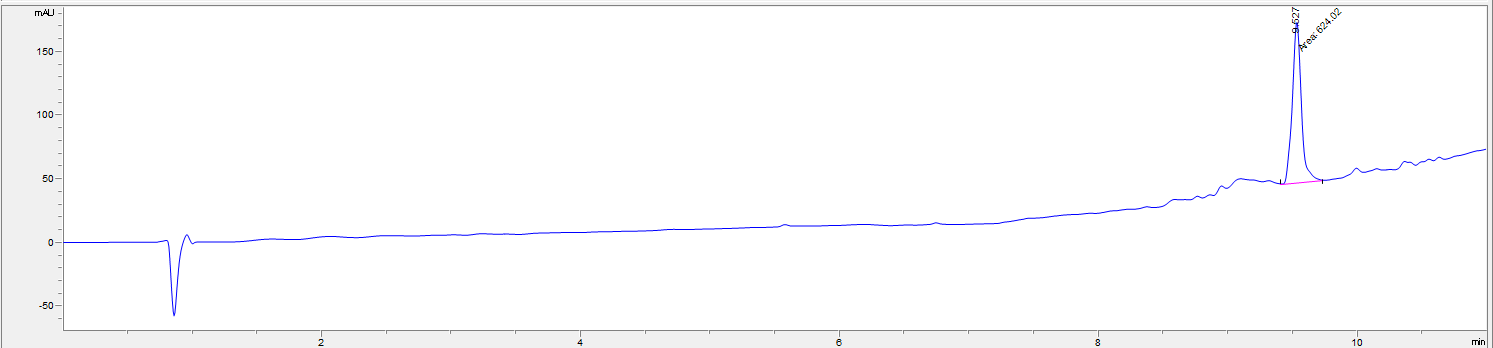


**Figure S10.** HPLC*-*DAD chromatogram obtained for lake natural water sample (*λ* = 220 nm, *C*0 (ibuprofen) = 20.63 mg L**^−^**1, *C*f (ibuprofen) = 23.5 mg L**^−^**1).


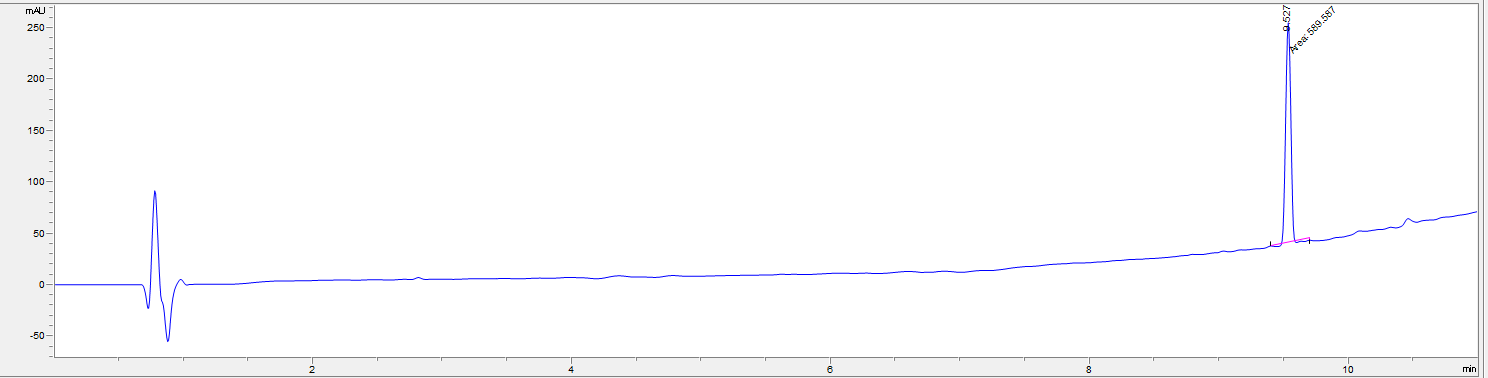


**Figure S11.** HPLC*-*DAD chromatogram obtained for river natural water sample (*λ* = 220 nm, *C*0 (ibuprofen) = 20.63 mg L**^−^**1, *C*f (ibuprofen) = 22.1 mg L**^−^**1).


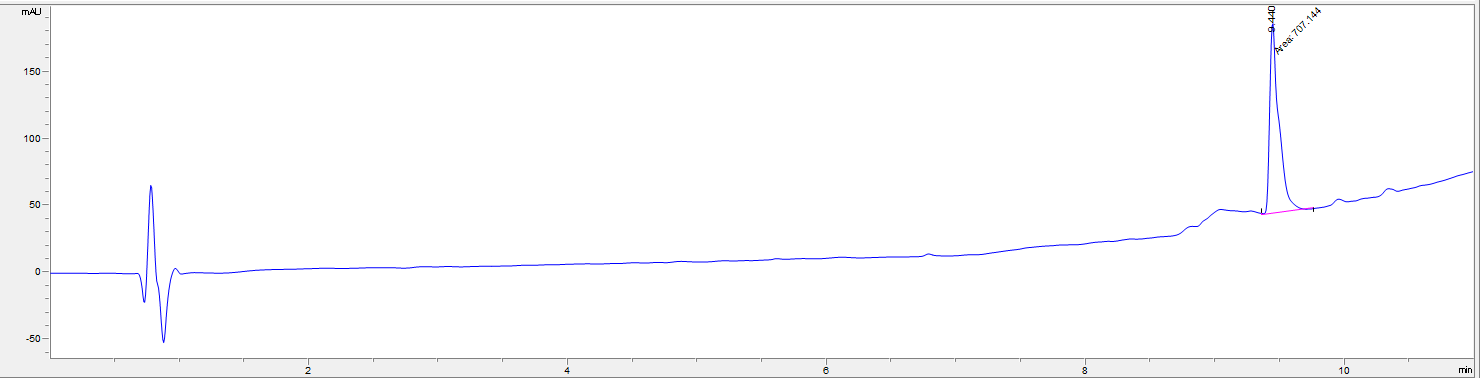


**Figure S12.** HPLC*-*DAD chromatogram obtained for estuary natural water sample (*λ* = 220 nm, *C*0 (ibuprofen) = 20.63 mg L**^−^**1, *C*f (ibuprofen) = 26.9 mg L**^−^**1).


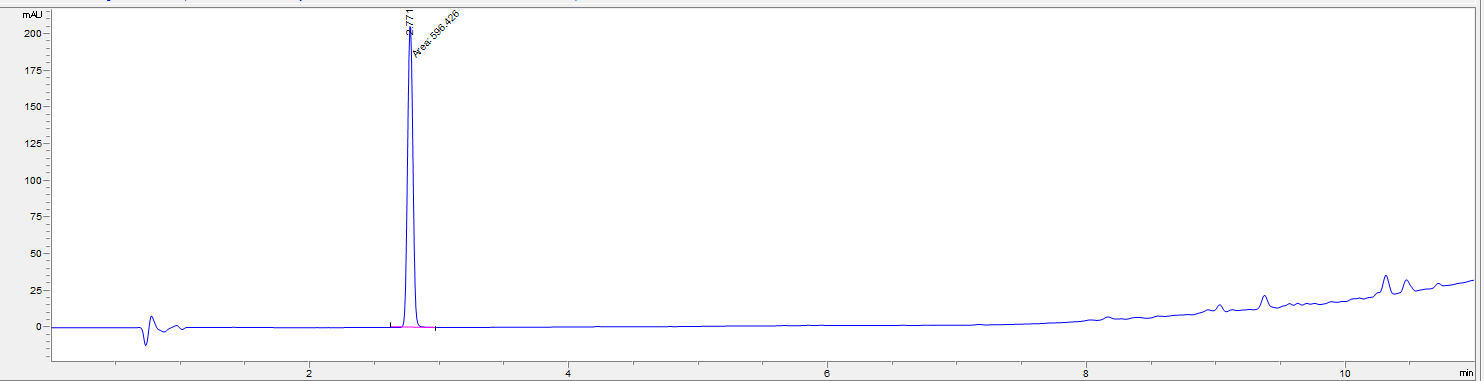


**Figure S13.** HPLC*-*DAD chromatogram obtained for lake natural water sample (*λ* = 243 nm, *C*0 (acetaminophen) = 15.12 mg L**^−^**1, *C*f (acetaminophen) = 10.5 mg L**^−^**1).


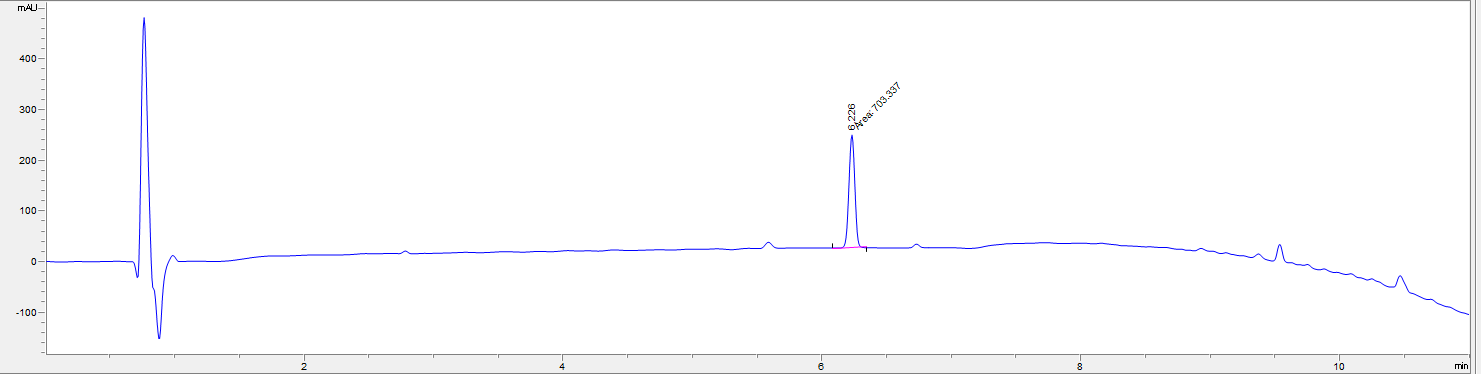


**Figure S14.** HPLC*-*DAD chromatogram obtained for lake natural water sample (*λ* = 210 nm, *C*0 (phenobarbital) = 34.84 mg L**^−^**1, *C*f (phenobarbital) = 35.7 mg L**^−^**1).

The chromatograms of natural water samples of lake, river, and estuary after adsorption experiments with COF were obtained running at the wavelengths of analysis for ibuprofen (*λ* = 220 nm, *t*r = 9.5 min), acetaminophen (*λ* = 243 nm, *t*r = 2.8 min), and phenobarbital (*λ* = 210 nm, *t*r = 6.1 min). *C*0 corresponds to the concentration of the pharmaceutical spiked in natural water samples; *C*f corresponds to the concentration of pharmaceutical observed in natural water samples after spiking, calculated using the calibration curve of each pharmaceutical.


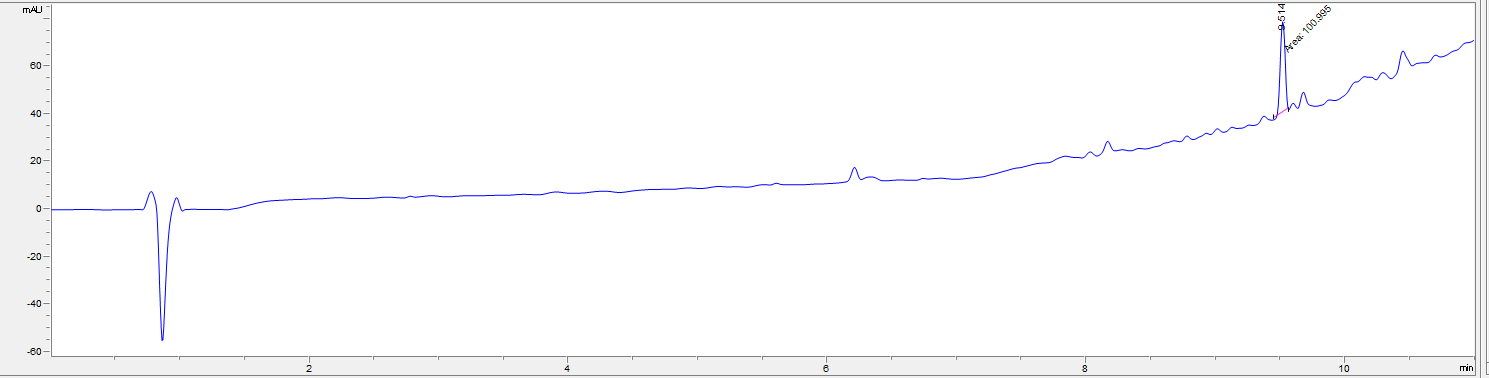


**Figure S15.** HPLC*-*DAD chromatogram obtained for lake natural water sample (*λ* = 220 nm, *C*0 (ibuprofen) = 0.10 mmol L^−^1).


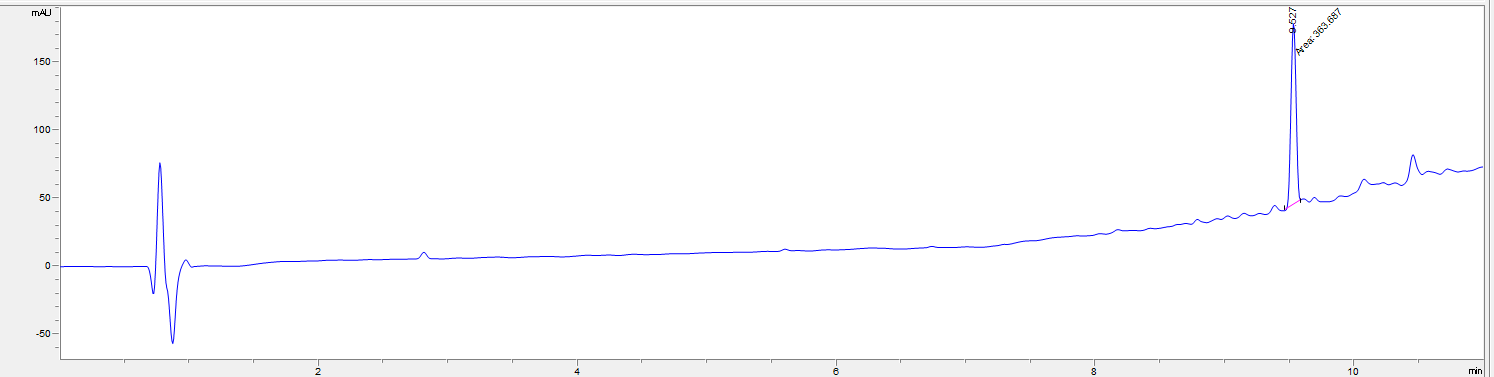


**Figure 16.** HPLC*-*DAD chromatogram obtained for river natural water sample (*λ* = 220 nm, *C*0 (ibuprofen) = 0.10 mmol L^−^1).


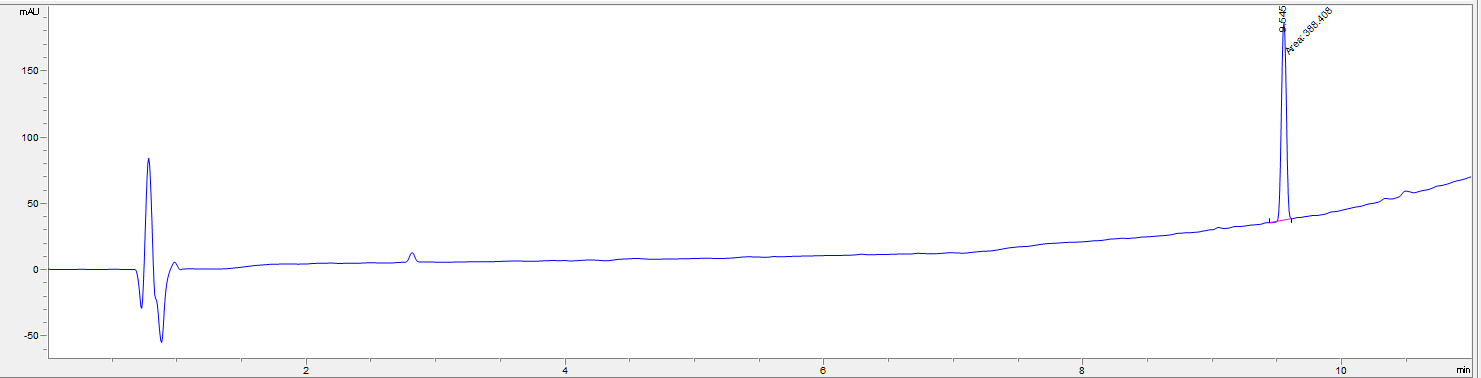


**Figure 17.** HPLC*-*DAD chromatogram obtained for estuary natural water sample (*λ* = 220 nm, *C*0 (ibuprofen) = 0.10 mmol L^−^1).


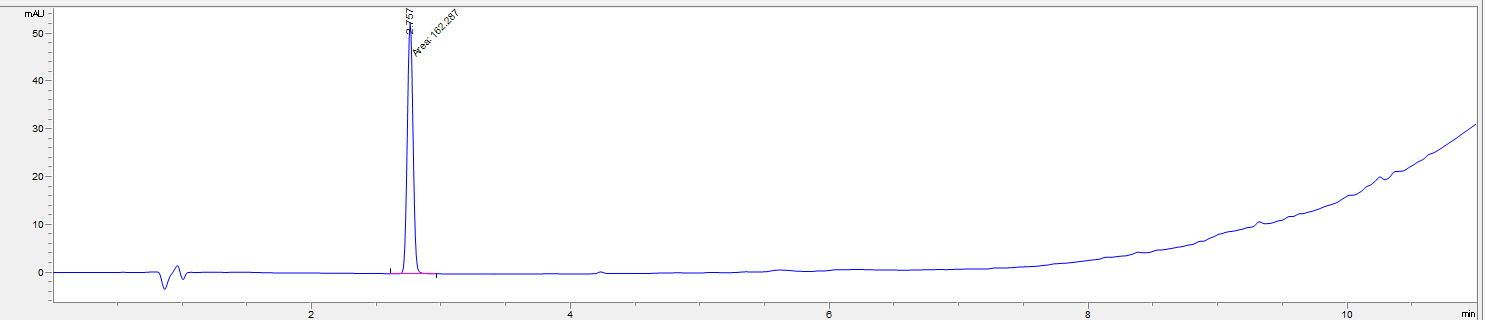


**Figure S18.** HPLC*-*DAD chromatogram obtained for lake natural water sample (*λ* = 243 nm, *C*0 (acetaminophen) = 0.15 mmol L^−^1).


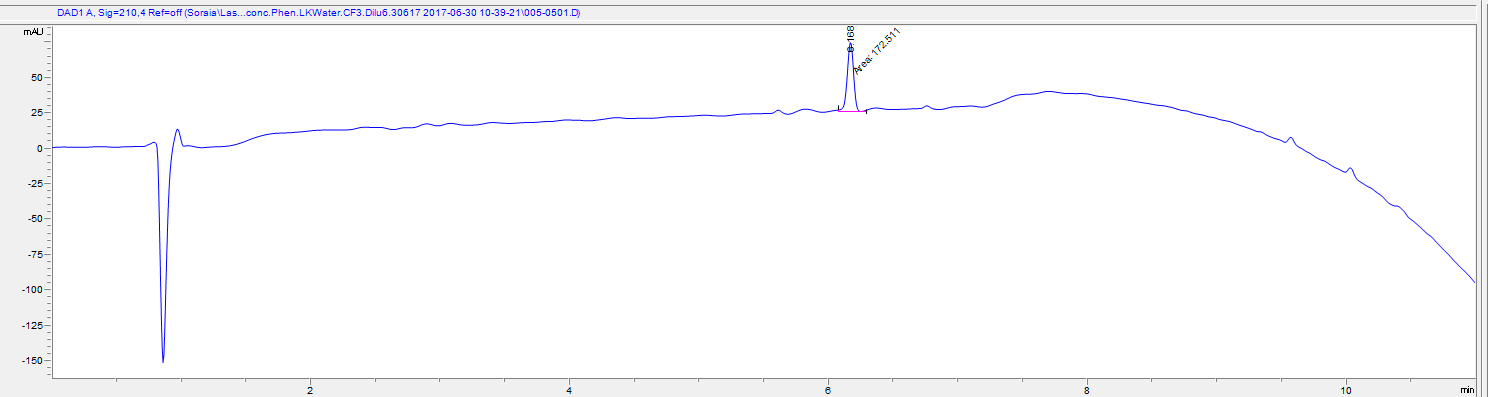


**Figure S19.** HPLC*-*DAD chromatogram obtained for lake natural water sample (*λ* = 210 nm, *C*0 (phenobarbital) = 0.15 mmol L^−^1).

8. Calibration Curves of Ibuprofen, Acetaminophen, and Phenobarbital

To all curves a non-measured point at 0,0 was added.

**Figure S20.** Calibration curve of ibuprofen (*λ* = 220 nm) at 21 °C in ultrapure water at pH 6–7.

**Figure S21.** Calibration curve of acetaminophen (*λ* =243 nm) at 21 °C in ultrapure water at pH 6–7.

**Figure S22.** Calibration curve of phenobarbital (*λ* =210 nm) at 21 °C in ultrapure water at pH 6–7.

9. Theoretical Calculations


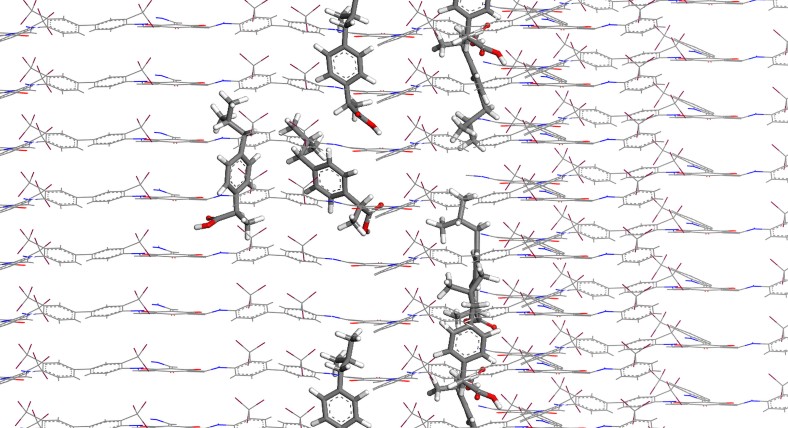


**Figure S23.** A view perpendicular to the COF pore axis showing the protonated ibuprofen in tilted and parallel orientation with respect to the COF pore axis.


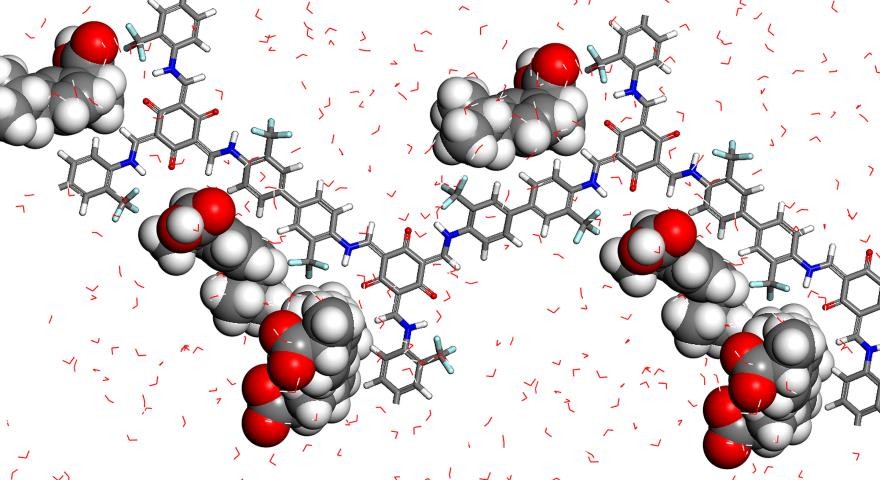


**Figure S24.** A view along the COF pore axis showing the orientations of deprotonated and protonated ibuprofen molecules in the model in a water environment. As a 1:1 mixture, the ibuprofen guests feature much less stable orientations with respect to the COF as compared to the individual models, resulting in a disordered arrangement of the guests in the COF pore alternating between the position found in vacuum and a tilted orientation relative to the COF pore axis.

References:

1. Chong, J.H.; Sauer, M.; Patrick, B.O.; MacLachlan, M.J. Highly Stable Keto-Enamine Salicylideneanilines. *Org. Lett.* **2003**, *5*, 3823–3826, doi:10.1021/ol0352714.
2. Mellah, A.; Fernandes, S.P.S.; Rodríguez, R.; Otero, J.; Paz, J.; Cruces, J.; Medina, D.D.; Djamila, H.; Espiña, B.; Salonen, L.M. Adsorption of Pharmaceutical Pollutants from Water Using Covalent Organic Frameworks. *Chem. Eur. J.* **2018**, *24*, 10601–10605, doi:10.1002/chem.201801649.
